# Evaluation of CE-MATRIX-Enhanced FLAIR imaging in the detection of leptomeningeal metastasis

**DOI:** 10.1186/s40644-025-00867-z

**Published:** 2025-04-08

**Authors:** Junhui Yuan, Shaobo Fang, Fan Meng, Yue Wu, Dongqiu Shan, Chunmiao Xu, Renzhi Zhang, Xuejun Chen

**Affiliations:** 1 Department of Medical Imaging, The Affiliated Cancer Hospital of Zhengzhou University & Henan Cancer Hospital, Zhengzhou, Henan Province 450008 China; 2 Department of Radiology, The First Affiliated Hospital of Fujian Medical University, 20 Cha-Zhong Road, Fuzhou, Fujian 350005 People’s Republic of China; 3 Department of Radiology, Academy of Medical Sciences, Zhengzhou University People’s Hospital & Henan Provincial People’s Hospital, Zhengzhou University, Zhengzhou, Henan Province 450008 China; 4 National Clinical Research Center for Cancer/Cancer Hospital, National Cancer Center, Chinese Academy of Medical Sciences and Peking Union Medical College, Beijing, 100021 China

**Keywords:** Magnetic resonance imaging, Leptomeningeal metastasis, Black blood sequence, Fluid attenuated inversion recovery sequence

## Abstract

**Objective:**

To investigate the diagnostic value of CE-MATRIX-T1FLAIR and 3D CE-T2FLAIR sequences based on Contrast Enhancement Modulated flip Angle Technique in Refocused Imaging with eXtended echo train (CE-MATRIX) technology for detecting Leptomeningeal Metastasis (LM) using Fluid Attenuated Inversion Recovery (FLAIR) imaging.

**Methods:**

This prospective study included 563 hospitalized patients with clinically suspected LM, diagnosed with malignant tumors between January 2022 and October 2023 at Henan Cancer Hospital. Both CE-MATRIX-T1FLAIR and 3D CE-T2FLAIR sequences were used for imaging. Two radiologists independently evaluated image quality, diagnostic confidence, and objective measurements, diagnosing LM as positive or negative, with disagreements resolved by consultation. Subjective and objective scores were compared using the Wilcoxon signed-rank test. The diagnostic performance of the sequences was compared using ROC curve analysis, with cerebrospinal fluid (CSF) cytology as the gold standard. Sensitivity, specificity, positive predictive value (PPV), negative predictive value (NPV), accuracy, and area under the curve (AUC) values were calculated and compared using Z-tests.

**Results:**

LM was confirmed in 321 patients. CE-MATRIX-T1FLAIR showed superior subjective scores in image quality and diagnostic confidence (*p* < 0.001). Though CE-MATRIX-T1FLAIR had a lower SNR (*p* = 0.013), it demonstrated higher sensitivity, specificity, PPV, NPV, accuracy, and AUC than 3D CE-T2FLAIR (*p* < 0.001). Both sequences provided effective diagnosis and differentiation of LM.

**Conclusion:**

CE-MATRIX-T1FLAIR offers superior diagnostic performance compared to 3D CE-T2FLAIR for LM, with slightly better subjective ratings despite a lower SNR. Both sequences are effective for diagnosing LM.

## Background

Leptomeningeal Metastasis (LM) has long been regarded as a late complication of solid malignancies, with persistent treatment challenges that have not seen significant progress over the decades [1, 2]. The disease state has historically resulted in severe neurological morbidity and rapid mortality, largely due to the prevalent treatment nihilism and the limited consensus among experts regarding optimal diagnostic and therapeutic strategies [3].

Currently, cerebrospinal fluid (CSF) cytology remains the gold standard for diagnosing leptomeningeal metastasis (LM). However, this method is prone to false negatives, with tumor cell detection rates ranging from 60.5 to 83% [4]. Furthermore, the diagnostic sensitivity of standard cytological techniques is reported to vary between 50% and 90%, as noted by Neuro-Oncology and the American Society of Clinical Oncology [5]. With the advancement of MRI technology, particularly in visualizing the subarachnoid space, imaging has become the primary, often sole, diagnostic tool. Numerous case series of LM have been reported, though only a few have been conducted during the era of high-quality MRI technology [2]. Currently, contrast-enhanced MRI with FLAIR sequences is considered the preferred imaging method for detecting and diagnosing LM. The European Association for Neuro and European Society for Medical Oncology (EANO-ESMO) clinical practice guidelines for the diagnosis, treatment, and follow-up of solid tumor leptomeningeal metastasis propose that enhanced 3D-FLAIR sequences should be prioritized for diagnosing LM [6].

The three-dimensional Fast Spin Echo (3D FSE) black-blood sequence, utilizing the modulated flip-angle technique in refocusing imaging with extended echo trains (MATRIX), employs an iterative method to optimize protocols from an inversion angle database, effectively suppressing blood flow signals within vessels [7]. Compared to traditional FSE sequences, the MATRIX sequence accelerates imaging by using longer echo train lengths (ETL) and shorter echo spacing (ESP). Studies have shown that the MATRIX sequence can reduce the acquisition time for routine clinical knee MRI without compromising image quality, signal-to-noise ratio (SNR), or contrast-to-noise ratio (CNR) [8]. However, there are few reports of its application in the detection and diagnosis of leptomeningeal metastasis.

This study aims to explore the diagnostic value of CE-MATRIX technology, specifically the Fluid Attenuated Inversion Recovery (FLAIR) T1WI (CE-MATRIX-T1FLAIR) and T2WI (3D CE-T2FLAIR) sequences, for detecting and diagnosing LM.

## Methods

**Fig. 1:**
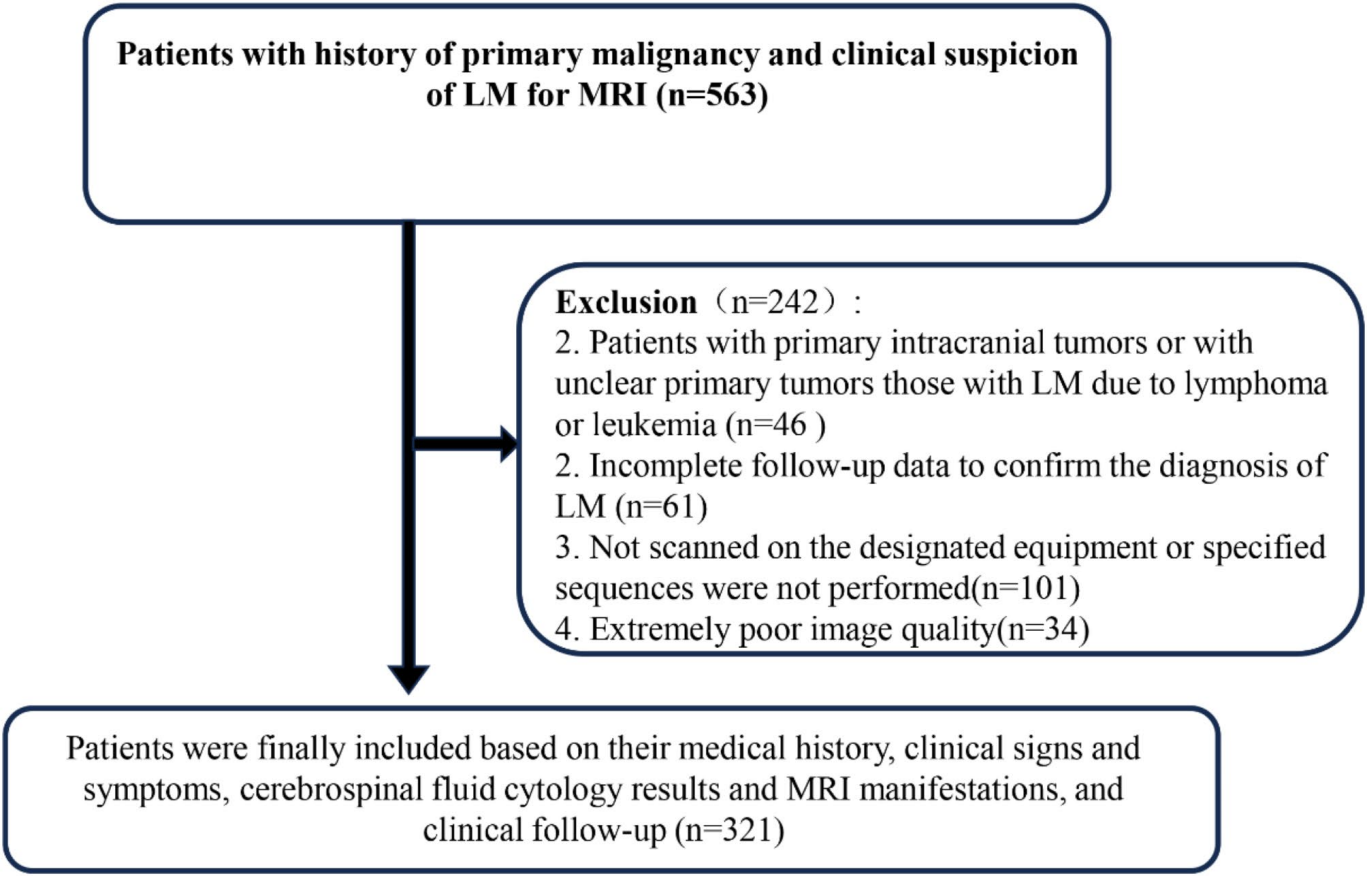
Patient flow chart

This study was approved by the Medical Ethics Committee of Henan Cancer Hospital (Approval No. 2022-KY-0063-001), with an exemption from obtaining informed consent. A prospective, consecutive cohort of 563 patients was enrolled between January 2021 and October 2023 at Henan Cancer Hospital. These patients were diagnosed with primary malignant tumors by pathology and clinically suspected of having leptomeningeal metastasis (LM), all of whom underwent MRI scans. Exclusion criteria: Patients with primary intracranial tumors or those with unclear primary tumors, as well as LM caused by lymphoma or leukemia; Patients whose LM diagnosis could not be confirmed based on clinical history, symptoms, cerebrospinal fluid (CSF) cytology results, MRI findings, and clinical follow-up; Patients who did not undergo MRI scans on the designated equipment or failed to undergo the designated MRI sequences; Patients with extremely poor image quality. A total of 321 patients were ultimately included in the study, consisting of 116 males and 205 females, aged 12 to 88 years (mean age: 57 ± 11 years). The primary tumor sources were as follows: 209 cases of lung cancer, 93 cases of breast cancer, 5 cases of colorectal cancer, 3 cases of liver cancer, 3 cases of esophageal cancer, 2 cases of gastric cancer, 1 case of osteosarcoma, 1 case of nasopharyngeal carcinoma, 1 case of submandibular gland cancer, 1 case of mediastinal neuroendocrine cancer, 1 case of thymic cancer, and 1 case of left lower limb osteosarcoma. The inclusion and exclusion criteria for the study are shown in Fig. 1.

### MRI protocal

The scans were conducted using a China United Imaging 3.0T uMR770 scanner equipped with a 16-channel head and neck coil, covering the entire brain. For the MRI contrast-enhanced imaging, a high-pressure injector was used to administer the gadopentetate dimeglumine contrast agent (General Electric Pharmaceutical Co., Ltd.) via an elbow vein, at a dose of 0.1 mmol/kg and a flow rate of 2.5 ml/s.

The scanning sequences and parameters are as follows:

- CE-MATRIX-T1FLAIR: TR = 650 ms, TE = 8.2 ms, TI = 850 ms, slice thickness = 1 mm, interslice gap = 0 mm, scan time = 300 s.
- 3D CE-T2FLAIR: TR = 3000 ms, TE = 406 ms, TI = 923 ms, slice thickness = 1 mm, interslice gap = 0 mm, scan time = 132 s.

### Diagnosis of LM

The diagnostic gold standard for LM is established based on the EANO-ESMO guidelines [2]. According to these criteria, LM diagnosis is classified into three categories: Type I (confirmed cases with positive cerebrospinal fluid cytology or pathological findings), Type II (cases with characteristic MRI features accompanied by clinical symptoms), and Type III (cases with either isolated clinical or neuroimaging evidence). To ensure the reliability of the gold standard, only Type I cases were designated as true positives. Type II and Type III cases were included in the analysis only after confirmation through follow-up assessments.

### Image analysis

Subjective Scoring: all images were uploaded to the United Imaging uWS-MR (version R005) system post-processing workstation for analysis. Two radiologists with over 10 years of diagnostic experience independently evaluated the image quality and diagnostic confidence of the CE-MATRIX-T1FLAIR and 3D CE-T2FLAIR images using a double-blind method. They also provided a diagnosis of LM (positive or negative), with any uncertainties resolved through consensus between the two radiologists. The results of LM were not known during the evaluation. T1 and T2 FLAIR were for the same patient. LM Diagnosis Criteria:

1. Linear or fluffy enhancement extending into the sulcal folds of the brain, which may be continuous or focal, and may appear as nodular. These lesions are commonly located on the surface of the cerebral hemisphere, the basement pool, the cerebellar tentorium, and the ependymal surface of the ventricles.
2. Enhancement or thickening of cranial (spinal) nerves.
3. Presence or absence of surrounding brain parenchymal edema or hydrocephalus manifestations such as ventricular dilation.

The image quality was assessed using the Likert scale with a 5-point system:

- 1 point: Significant artifacts, unable to diagnose.
- 2 points: Moderate artifacts, prone to misdiagnosis.
- 3 points: Minimal artifacts, minor impact on diagnosis.
- 4 points: Few artifacts, diagnosis can be clearly made.
- 5 points: No artifacts, diagnosis can be clearly made.

Diagnostic confidence was rated using the Likert scale with a 4-point system:

- 1 point: Unable to diagnose.
- 2 points: Diagnosis difficult.
- 3 points: Marginal diagnosis.
- 4 points: Clear diagnosis.

**Fig. 2:**
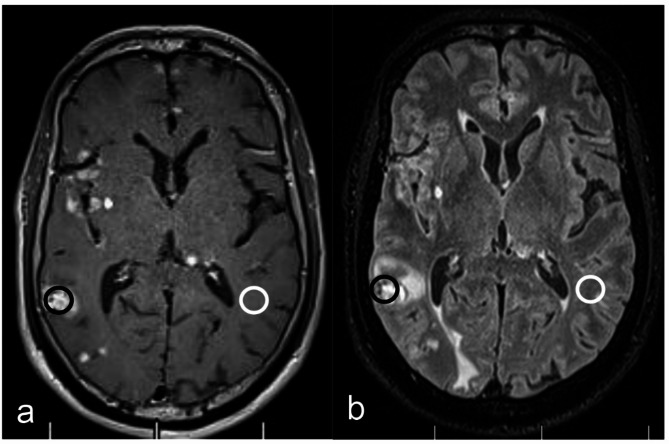
Schematic diagrams of CE-MATRIX-T1FLAIR and 3D CE-T2FLAIR tumor area and contralateral brain white matter ROIs. (**a**) CE-MATRIX-T1FLAIR plot, black circle is ROI of tumor area, white circle is ROI of contralateral brain white matter area. (**b**) 3D CE-T2FLAIR image, black circle is the ROI of the tumor region, white circle is the ROI of the contralateral white matter region

Objective Measurement: two radiologists, with 10 (Radiologist 1) and 15 (Radiologist 2) years of diagnostic experience, independently delineated regions of interest (ROI) of 20–55 mm² in the LM-enhanced area and the contralateral normal white matter in both the CE-MATRIX-T1FLAIR and 3D CE-T2FLAIR images (Figs. 2). The measurements were taken three times, and the average value was calculated. The signal intensity (SI) and standard deviation (SD) of each ROI were measured, and the signal-to-noise ratio (SNR) and contrast-to-noise ratio (CNR) were calculated and compared using the following formulas:

- SNR = SI (tumor) / SD (white matter).
- CNR = (SI (tumor) – SI (white matter)) / SD (white matter).

Where SD represents the noise, or the standard deviation of the SI within the ROI.

### Statistical analysis

Statistical analysis was performed using SPSS 26.0 software. The consistency of inter-observer subjective scores and objective measurements was evaluated using the Kappa test and intra-class correlation coefficient (ICC). The following criteria were used to interpret consistency:

- Kappa/ICC ≤ 0.2: Poor consistency.
- 0.2 < Kappa/ICC ≤ 0.4: Fair consistency.
- 0.4 < Kappa/ICC ≤ 0.6: Moderate consistency.
- 0.6 < Kappa/ICC ≤ 0.8: Good consistency.
- Kappa/ICC > 0.8: Excellent consistency.

Data conforming to a normal distribution are presented as c ± s, while non-normally distributed data are presented as median (interquartile range). The Wilcoxon signed-rank test was used for inter-group comparisons of subjective scores (image quality, diagnostic confidence) and objective scores.

Cerebrospinal fluid cytological results were used as the gold standard for diagnosis. The diagnostic efficacy, sensitivity, specificity, positive predictive value, negative predictive value, and accuracy of the CE-MATRIX-T1FLAIR and 3D CE-T2FLAIR sequences for diagnosing LM were compared using ROC curve analysis. A Z-test was used to compare the diagnostic efficacy between the two sequences. Statistical significance was defined as *P* < 0.05.

## Results

### Consistency test

The consistency of the subjective scores and objective measurements (tumor region and contralateral white matter SI and SD values) between the CE-MATRIX-T1FLAIR and 3D CE-T2FLAIR sequence images showed good agreement, with ICC/Kappa values ranging from 0.76 to 0.85 (*p* < 0.05). The results evaluated by Radiologist 2 were used for further data analysis.

### Comparison of subjective scores (Image quality, diagnostic Confidence) and objective measurements between the two sequences

**Fig. 3:**
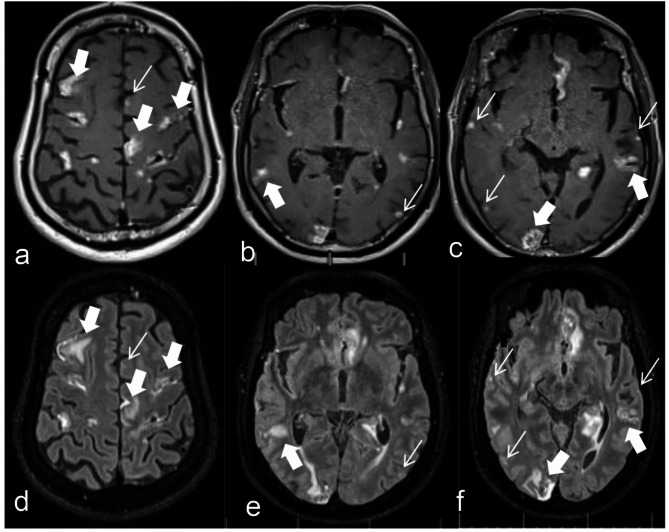
Comparative imaging of the LM using CE-MATRIX-T1FLAIR (**a-c**) and 3D CE-T2FLAIR (**d-f**). The CNR of 3D CE-T2FLAIR image is slightly better than that of CE-MATRIX-T1FLAIR, but the extent of the lesion cannot be accurately displayed due to the image of brain parenchyma edema around the lesion (indicated by the thick arrows), and the lesion extent of pilonidal metastasis is slightly larger in the same level of lesion (indicated by the thick arrows), and the lesion had a better contrast with the brain parenchyma. In addition CE-MATRIX-T1FLAIR has a higher detection rate of small lesions (indicated by thin arrows), which are more likely to be missed by 3D CE-T2FLAIR (indicated by thin arrows)

The Wilcoxon signed-rank test was used for inter-group comparisons of subjective scores (image quality, diagnostic confidence) and objective measurements. The subjective scores (image quality and diagnostic confidence) by the two radiologists for the CE-MATRIX-T1FLAIR sequence were higher than those for the 3D CE-T2FLAIR sequence, with *p* < 0.001, indicating statistically significant differences. The SNR of the CE-MATRIX-T1FLAIR sequence was lower than that of the 3D CE-T2FLAIR sequence, with *p* = 0.013, indicating a statistically significant difference. There was no statistically significant difference in CNR between the two sequences, with *p* = 0.618 (Table 1; Fig. 3).

**Table 1:** Subjective scores (image quality, diagnostic confidence) and objective measures

	CE-MATRIX-T_1_FLAIR	3D CE-T2FLAIR	*P* value
Subjective			
Image Quality	5(5, 5)	5(4, 5)	**< 0.001**
Diagnostic Confidence	4 (4, 4)	4 (3, 4)	**0.008**
Objective			
SNR	1.795 ± 0.828	3.451 ± 1.121	**0.013**
CNR	1.256 ± 0.321	1.309 ± 0.765	0.618

SNR, signal-to-noise ratio; CNR, contrast-to-noise ratio

### Comparison of diagnostic efficacy for LM between the two sequences

**Fig. 4:**
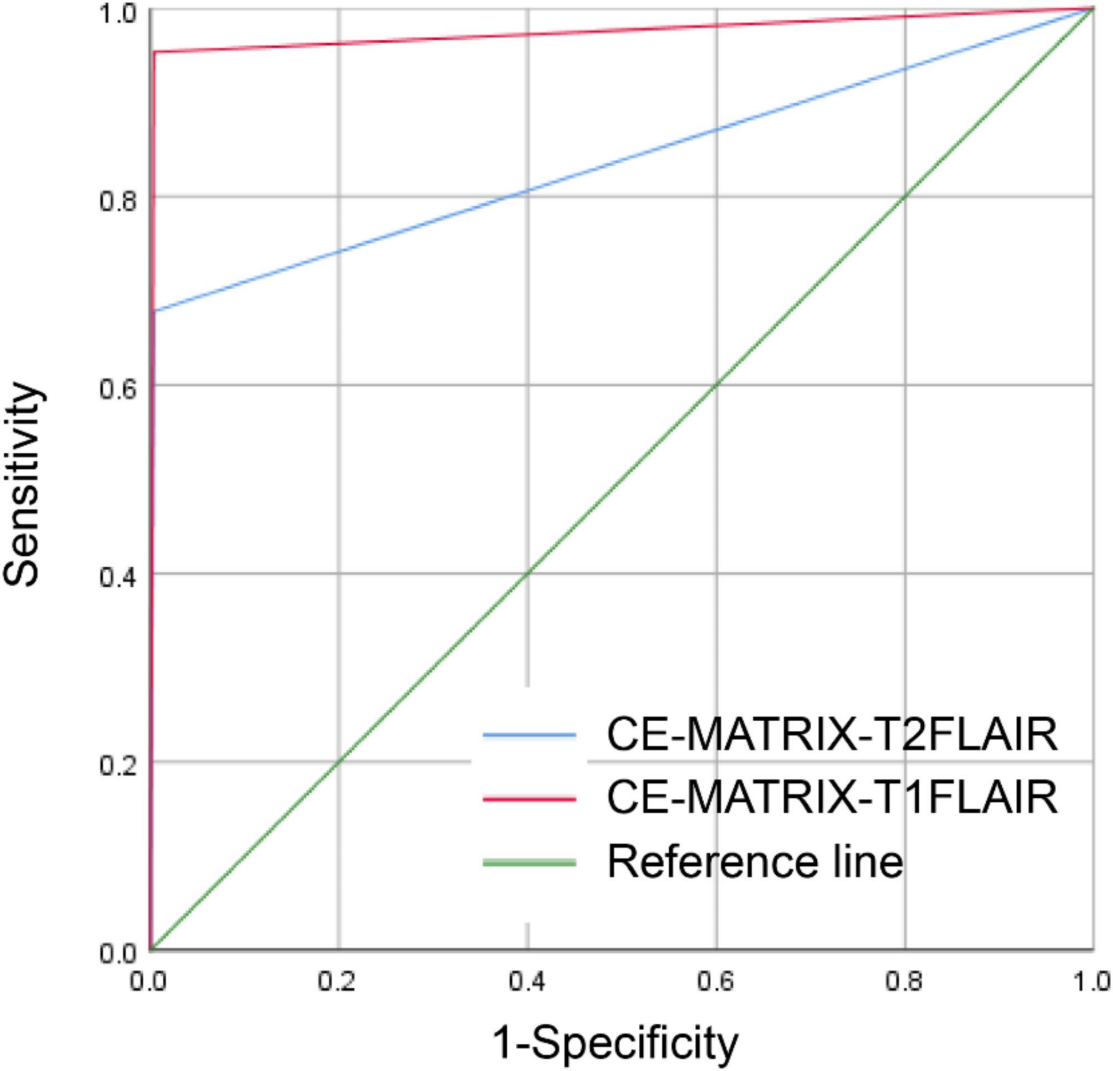
ROC curves for two sequences to detect and diagnose LMs

Using the patient’s medical history, clinical symptoms and signs, cerebrospinal fluid cytological results, MRI findings, and the final clinical follow-up results as the gold standard, ROC curve analysis was performed to compare the diagnostic efficacy of the two sequences for diagnosing LM. The area under the curve (AUC) of CE-MATRIX-T1FLAIR was higher than that of 3D CE-T2FLAIR. The sensitivity, positive predictive value, negative predictive value, and accuracy for diagnosing LM with the CE-MATRIX-T1FLAIR sequence were higher than those for the 3D CE-T2FLAIR sequence. Both sequences exhibited high and similar specificity. A Z-test was used to compare the diagnostic efficacy between the two sequences, and the Z-value was − 4.75 (*p* < 0.001), indicating a statistically significant difference (Table 2; Fig. 4).

**Table 2:** Comparison of the diagnostic efficacy of CE-MATRIX-T1FLAIR and 3D CE-T2FLAIR for the diagnosis of LM

Sequence	Sensitivity	Specificity	Positive Predictive value	Negative Predictive value	Accuracy	AUC	Z value	*P* value
CE-MATRIX-T_1_FLAIR	0.953	0.996	0.997	0.942	0.972	0.975	-4.75	**< 0.001**
3D CE-T2FLAIR	0.678	0.996	0.995	0.701	0.815	0.837		

AUC, area under the curve

## Discussion

The MATRIX sequence, based on variable flip angle technology and the 3D FSE black-blood sequence, utilizes iterative methods to modulate the optimal scheme in the inversion angle database, effectively suppressing the blood flow signals within blood vessels. This results in enhanced SNR and black-blood capability, allowing for clearer visualization of vessel walls, black blood flow backgrounds, and abnormal thrombus signals within the lumen. The sequence also offers the advantage of thin slice continuous scanning and multi-plane reconstruction. While the MATRIX sequence has been applied in areas such as deep vein thrombosis, cartilage injury diagnosis, and brain parenchymal metastases, its application for the detection and diagnosis of LM remains rarely reported. In this study, we found that both CE-MATRIX-T1FLAIR and 3D CE-T2FLAIR sequences exhibit high diagnostic efficacy for LM. Although the SNR of CE-MATRIX-T1FLAIR images was lower than that of 3D CE-T2FLAIR, the subjective scores (image quality and diagnostic confidence) and diagnostic efficacy of CE-MATRIX-T1FLAIR were slightly superior to 3D CE-T2FLAIR. Both sequences were effective for detecting, diagnosing, and differentiating LM.

The application of advanced MRI sequences, especially 3D FSE T1 black-blood imaging, has significantly improved the diagnosis and evaluation of LM, a severe complication of cancer. Black-blood MRI is renowned for suppressing vascular signals, allowing for better visualization of the meninges and tumor spread, thereby improving diagnostic accuracy. The ability of black-blood sequences to suppress blood vessel signals and reduce imaging artifacts has been proven to enhance the diagnostic sensitivity and specificity for LM. Sohn et al. [8] emphasized the effectiveness of 3D FSE T1 black-blood imaging in diagnosing and predicting the prognosis of leptomeningeal carcinomatosis. Their findings align with our study, highlighting the superior spatial resolution and contrast of black-blood imaging for visualizing tumor lesions in the cerebrospinal fluid (CSF) spaces. This technique has been shown to be particularly valuable in clinical decision-making, as it provides clear images of tumor margins and the extent of disease.

Furthermore, Kim et al. [9] demonstrated that black-blood imaging can detect smaller and early metastases, which are often overlooked in conventional MRI. Our study corroborates their findings, showing that CE-MATRIX-T1FLAIR is more effective at detecting micro-metastases and differentiating tumor lesions from surrounding tissues. Early detection of such lesions is crucial for improving patient prognosis through targeted interventions.

Sawano et al. [10] discussed the advantages of black-blood imaging in reducing vascular artifacts and improving soft tissue visibility, which enhances the delineation of tumors. Our findings extend their conclusions by showing that CE-MATRIX-T1FLAIR outperforms 3D CE-T2FLAIR in diagnostic accuracy and subjective evaluations, supporting its clinical utility in LM imaging. Additionally, combining black-blood imaging with other advanced modalities, such as contrast-enhanced MRI and functional imaging, can further enhance diagnostic performance. Honda et al. [11] demonstrated that integrating black-blood imaging with other sequences improves the visualization of tumor boundaries and structural assessment, which is critical for surgical planning and treatment. Our results align with these findings, emphasizing the potential of CE-MATRIX-T1FLAIR to provide comprehensive and accurate diagnostic information.

Li et al. [6] found that for knee joint MRI, the MATRIX sequence had high consistency in evaluating cartilage, subchondral bone, and ligaments compared to 2D FSE or PD sequences. Sui et al. [7] demonstrated that the MATRIX sequence, as a novel MR black-blood imaging sequence, reduces scan time by 30% without compromising image quality or diagnostic performance compared to conventional 2D sequences. Our study shows that both CE-MATRIX-T1FLAIR and 3D CE-T2FLAIR sequences exhibit excellent consistency in subjective evaluation and objective measurements (SI and SD values of tumor and contralateral white matter), with ICC/Kappa values ranging from 0.76 to 0.85. Compared to 3D CE-T2FLAIR, CE-MATRIX-T1FLAIR demonstrated statistically significant advantages in subjective evaluations of image quality and diagnostic confidence. However, CE-MATRIX-T1FLAIR’s SNR was slightly lower than that of 3D CE-T2FLAIR. Nonetheless, the difference in CNR between the two sequences was not statistically significant. In our study, the lower SNR of CE-MATRIX-T1FLAIR compared to CE-T2FLAIR may be due to sequence-related factors. In T2-weighted imaging, a longer echo time (TE) enhances T2 contrast by allowing more time for transverse magnetization decay, increasing signal intensity. T2-FLAIR, as a fluid-attenuated sequence, suppresses free water signals but is less effective on bound water with different longitudinal relaxation times. This can cause certain white matter hyperintensities to persist on CE-T2FLAIR, making them harder to distinguish from leptomeningeal disease, potentially leading to misdiagnosis.

When comparing the diagnostic efficacy for LM, CE-MATRIX-T1FLAIR showed a higher AUC in ROC analysis, indicating better overall performance. CE-MATRIX-T1FLAIR exhibited higher sensitivity, positive predictive value (PPV), negative predictive value (NPV), and accuracy compared to 3D CE-T2FLAIR, while both sequences showed similar specificity.

However, the diagnosis of leptomeningeal metastasis (LM) is particularly challenging due to the small size and complex distribution of lesions. The fusion of multimodal imaging can significantly improve detection efficiency by combining the strengths of different imaging techniques. Diffusion Weighted Imaging (DWI) detects lesions with high cellular density by identifying restricted diffusion of water molecules. Perfusion MRI assesses the hemodynamic characteristics of the lesions, while the high-resolution 3D CE-MATRIX-T1FLAIR technique provides superior spatial resolution, enabling clear visualization of subtle meningeal enhancement. The combination of these three techniques helps to overcome each individual method’s limitations: DWI’s relatively low resolution, the limited sensitivity of perfusion MRI in lesions with poor blood supply, and CE-MATRIX-T1FLAIR’s ability to reveal fine meningeal enhancements that might be missed by other methods. Moreover, hybrid imaging techniques, such as combining CE-MATRIX with DWI, offer complementary anatomical and functional information. This combined approach significantly enhances both the sensitivity and specificity of LM detection. Future research should focus on further refining these hybrid imaging techniques.

In this study, we chose to compare CE-MATRIX-T1FLAIR with 3D CE-T2FLAIR based on the recommendations from the Chinese Integrated Oncology Guidelines—Brain Metastases and the European Association for Neuro-Oncology (EANO) and European Society for Medical Oncology (EANO-ESMO) clinical practice guidelines [4]. These guidelines indicate that contrast-enhanced 3D-FLAIR is the preferred sequence for LM diagnosis. Furthermore, multiple studies [12–14] have demonstrated that contrast-enhanced 3D-FLAIR has higher sensitivity than contrast-enhanced 3D-T1WI for LM diagnosis, which is why our study focuses on the optimization and comparison of this sequence.

Additionally, previous research [15] has shown that CE-T2-FLAIR has been used to evaluate various inflammatory and non-inflammatory neurological diseases in academic medical centers and has demonstrated high prevalence and specificity in differentiating LM. Therefore, our study focuses on optimizing the best sequence recommended by existing guidelines. Future research may further explore the relative advantages and cost-effectiveness of CE-MATRIX-T1FLAIR compared to other imaging sequences, such as contrast-enhanced 2D axial T1WI and 3D contrast-enhanced T1WI.

Despite promising results, our study does have limitations. First, the sample size was relatively small, and larger-scale studies are needed to confirm these findings. Second, further research is needed to explore the integration of black-blood imaging with other advanced imaging techniques, such as diffusion-weighted imaging (DWI) or perfusion imaging, to offer a more comprehensive diagnostic approach. Future studies should also evaluate the cost-effectiveness and clinical utility of these advanced sequences in routine practice.

## Conclusions

In conclusion, both CE-MATRIX-T1FLAIR and 3D CE-T2FLAIR can be used for detecting, diagnosing, and differentiating LM.

## Data Availability

No datasets were generated or analysed during the current study.

## Abbreviations

CE: MATRIX-Contrast Enhancement Modulated flip Angle Technique in Refocused Imaging with extended echo train
FLAIR: Fluid Attenuated Inversion Recovery
LM: Leptomeningeal Metastasis
CSF: Cerebrospinal Fluid
SNR: Signal-to-Noise Ratio
CNR: Contrast-to-Noise Ratio
PPV: Positive Predictive Value
NPV: Negative Predictive Value
AUC: Area Under the Curve
ROC: Receiver Operating Characteristic
MRI: Magnetic Resonance Imaging
ICC: Intra-Class Correlation
TR: Repetition Time
TE: Echo Time
ETL: Echo Train Length
ESP: Echo Spacing
ROI: Region of Interest
